# Differences in Clinical Outcomes of Adults Referred to a Homeless Transitional Care Program Based on Multimorbid Health Profiles: A Latent Class Analysis

**DOI:** 10.3389/fpsyt.2021.780366

**Published:** 2021-12-20

**Authors:** Colin M. Smith, Jacob Feigal, Richard Sloane, Donna J. Biederman

**Affiliations:** ^1^Department of Psychiatry and Behavioral Sciences, Duke University School of Medicine, Durham, NC, United States; ^2^Department of Medicine, Duke University School of Medicine, Durham, NC, United States; ^3^Center for the Study of Aging, Duke University Medical Center, Durham, NC, United States; ^4^Clinical Health Systems & Analytics Division, Duke University School of Nursing, Durham, NC, United States

**Keywords:** homelessness, medical respite, comorbidity, tri-morbidity, latent class analysis, mental health, substance use

## Abstract

**Background:** People experiencing homelessness face significant medical and psychiatric illness, yet few studies have characterized the effects of multimorbidity within this population. This study aimed to (a) delineate unique groups of individuals based on medical, psychiatric, and substance use disorder profiles, and (b) compare clinical outcomes across groups.

**Methods:** We extracted administrative data from a health system electronic health record for adults referred to the Durham Homeless Care Transitions program from July 2016 to June 2020. We used latent class analysis to estimate classes in this cohort based on clinically important medical, psychiatric and substance use disorder diagnoses and compared health care utilization, overdose, and mortality at 12 months after referral.

**Results:** We included 497 patients in the study and found 5 distinct groups: “low morbidity” (referent), “high comorbidity,” “high tri-morbidity,” “high alcohol use,” and “high medical illness.” All groups had greater number of admissions, longer mean duration of admissions, and more ED visits in the 12 months after referral compared to the “low morbidity” group. The “high medical illness” group had greater mortality 12 months after referral compared to the “low morbidity” group (OR, 2.53, 1.03–6.16; 95% CI, 1.03–6.16; *p* = 0.04). The “high comorbidity” group (OR, 5.23; 95% CI, 1.57–17.39; *p* < 0.007) and “high tri-morbidity” group (OR, 4.20; 95% CI, 1.26–14.01; *p* < 0.02) had greater 12-month drug overdose risk after referral compared to the referent group.

**Conclusions:** These data suggest that distinct groups of people experiencing homelessness are affected differently by comorbidities, thus health care programs for this population should address their risk factors accordingly.

## Introduction

Medical respite programs provide a safe place for recovery from illness or injury for people experiencing homelessness (PEH). A growing literature base describes the implementation and success of such programs for this population (1–3). Given that PEH face high rates of complex yet modifiable illness (4), and that medical, mental health, and substance use disorders are linked to high health care utilization (5, 6), there remains a need to understand the specific risks that multimorbid health profiles portend for this population.

Population segmentation using latent class analysis (LCA) is one method to tailor integrated health care interventions to groups facing high rates of multimorbidity with the aim of reducing negative clinical outcomes and unnecessary health care utilization (7). Broadly, LCA is a statistical method using maximum likelihood estimation to classify distinct subgroups of people within a population based on shared characteristics (8). The resulting subgroups, referred to as latent classes or groups, are internally homogeneous and externally heterogeneous (9). LCA has been used successfully to classify primary care utilizers into patient classes, which have predictive value in clinical outcomes (10), although fewer LCAs have focused on PEH.

Early LCAs of PEH were used to describe the typology of psychiatric illness (11), substance use (12), and medical illness (13) in this population. More recently, LCA has been used as a method to assess social (14–16) and health services (17, 18) utilization amongst distinct groups of PEH. An analysis of 2010 Medicaid claims data of PEH examined heterogeneity among frequent emergency department (ED) users in a single health care center and identified distinct subgroups in the LCA. Individuals with tri-morbid illness in this analysis had greater non-ED costs than other subgroups (17). A 2013 analysis of veterans experiencing homelessness used LCA based on risk factors for homelessness (e.g., presence of psychotic disorder, substance use disorder, post-traumatic stress disorder, low income, unemployment, incarceration, and chronic homelessness) and found differential homeless service utilization between individuals with dual diagnosis, abuse, incarceration, poverty, substance use disorder, and disabling medical problems (18).

However, such analyses of PEH have not assessed differential health outcomes based on class assignment (14–16) and often aggregate medical, psychiatric and substance use conditions as a single entity (17, 18). There are no published studies that classify individuals in medical respite or transitional care programs based on discrete medical, psychiatric, and substance use disorder profiles and compare clinical outcomes across these groups.

The primary objective of this retrospective cohort study was therefore to (a) estimate distinct classes in a cohort of adult PEH referred to a homeless transitional care program using LCA models, and (b) evaluate for differences in health care utilization and mortality across groups.

## Materials and Methods

### Study Design, Setting, and Participants

This study examined data from 497 adults referred to Durham Homeless Care Transitions (DHCT), a transitional care and medical respite program for PEH, from July 1, 2016 through June 30, 2020. DHCT inclusion criteria were adults who were (a) experiencing homelessness; (b) able to participate in and maintain a safe and harm-free environment; (c) willing to follow rules of housing setting, which may include abstinence from substances and alcohol; (d) willing to participate in case management visits and treatment plans; (e) competent in activities of daily living (self-toileting, simple meal preparation); (f) psychiatrically stable (e.g., no active threats of harm to self or others); and (g) cleared by physical therapy for home discharge when applicable. Data were collected and analysis was conducted between March 12, 2020 and May 27, 2021. The Duke Health Institutional Review Board approved all study procedures (Pro00103699).

### Data Collection

We extracted electronic health record (EHR) data from January 1, 2014, through June 30, 2020. We retrieved demographic data (i.e., sex, age, race, referral source, insurance), medical diagnoses (e.g., cardiovascular, chronic pulmonary, other end organ disease, diabetes), substance use diagnoses (e.g., alcohol use disorder, opioid use disorder, stimulant use disorder), and mental illness diagnoses (e.g., psychotic disorder, mood disorder, anxiety/trauma related disorders) according to ICD, Tenth Revision (ICD-10) codes (19–21) (see Supplementary 1). We chose these 10 diagnoses (risk factors) based on the high prevalence of such disorders amongst PEH (22, 23) and consensus of three individuals with expertise in providing medical and psychiatric care for PEH. We obtained encounter information (i.e., admissions, length of stay, ED visits, and outpatient visits within 12 months after program referral) and clinical outcomes (i.e., mortality and drug overdose at 12 months after program referral).

### Statistical Analyses

LCA statistical testing of model fit and class membership is probabilistic, with latent group membership probabilities computed for each subject from the set of candidate risk factors. For this LCA, the presence or absence of 10 risk factors at the referral date was used to define the latent groups: (a) substance use disorders (i.e., alcohol, opioid, and stimulants) (b) mental illness (i.e., psychotic disorder, anxiety/trauma, mood disorder), and (c) medical conditions (i.e., cardiovascular, diabetes, end organ, pulmonary). Following standard practice, we began with a one class model and then added classes in successive models (24). We used the Bayesian Information Criterion (BIC) to determine the optimal number of classes, as previous literature has shown BIC to be superior to other criteria at correctly identifying the number of groups in LCA processing, particularly when using categorical outcomes (24). In addition, we evaluated the resulting models for interpretability using clinical judgment (25). Each participant was assigned to their modal class based on the computed membership probabilities.

After defining the latent classes, our next analytical objective was to determine how the set of classifying variables (presence/absence of 10 risk factors) and demographic variables varied by latent class group using logistic regression or ordinary least square regression, as appropriate. To prevent overtesting, the utilization outcomes were evaluated using a referent latent group that was selected and compared to each of the other latent groups using Poisson regression.

The LCA was performed using the PROC LCA procedure in SAS v9.4 (26), which provides likelihood-based information indices to aid in assessing the number of latent classes needed to fit the data. To validate class selection, using simple random sampling, the 497 subjects were separated into two independent groups and the PROC LCA was conducted separately on each group. LCA for each sample resulted in an optimal 5-class solution. Zero-inflated Poisson (ZIP) regression was used to model the association of LCA group membership with the outcomes of medical utilization. Number of hospital admissions, emergency department encounters, and outpatient encounters are counts in nature, and the length of stay of admissions fit the Poisson distribution. The abundance of zero counts necessitated the use of the ZIP method. The occurrence of mortality and drug overdose were modeled using logistic regression. For both sets of analyses, the LCA classes were treated as nominal with “Low morbidity” as the referent group. The analyses were conducted using SAS version 9.4 (SAS Institute, Inc., Cary, NC).

## Results

### Selection of Latent Groups

**Figure 1:**
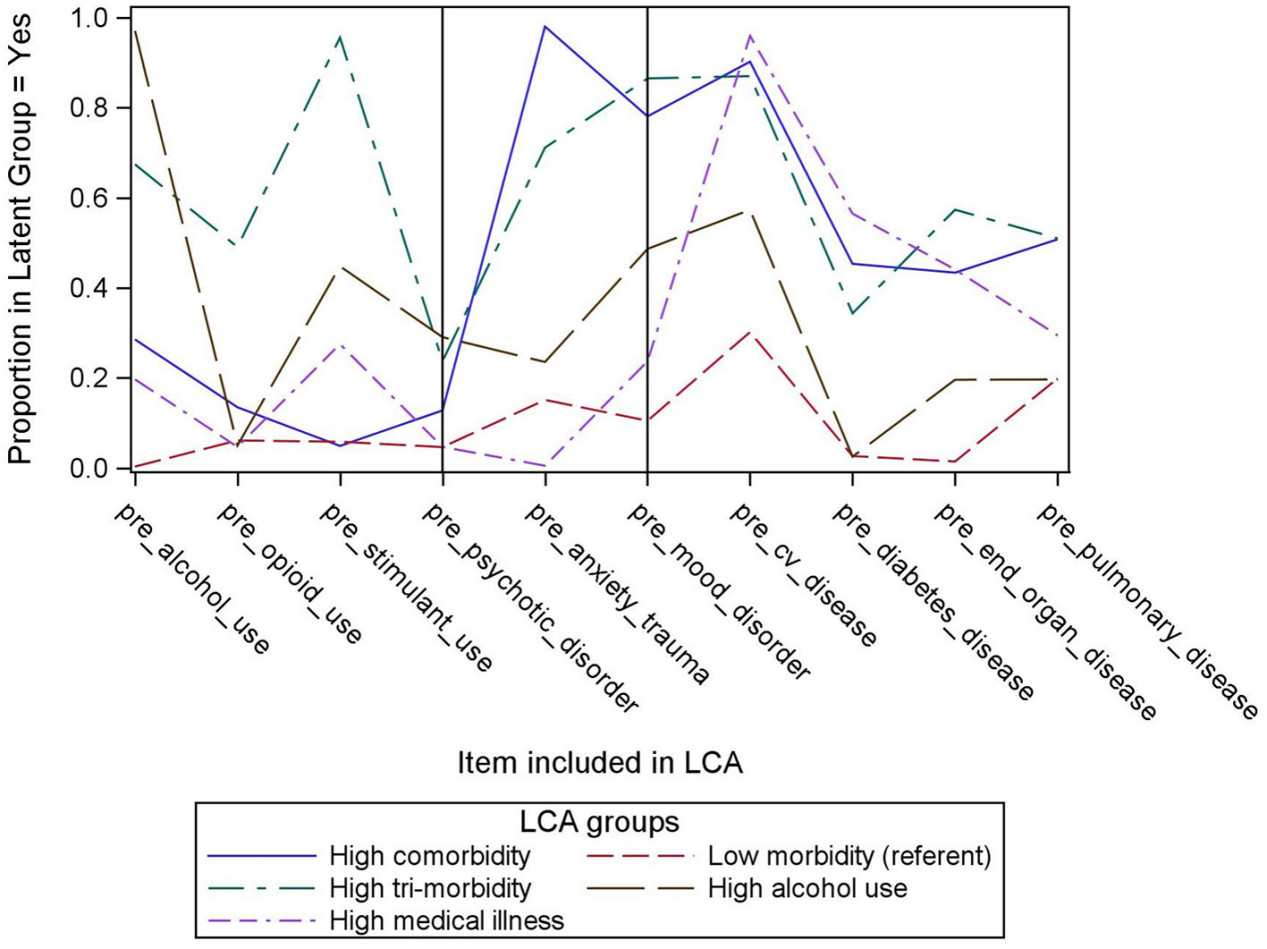
Five latent classes of adults referred to a homeless transitional care program.

A 5-group model, depicted in Figure 1, was chosen based on the optimal number of latent classes by comparing the BIC of the candidate models (25). The BIC of the five-class solution was 784.6 and the entropy was 0.87 (Supplementary 2). Figure 1 indicates the percent of subjects in each group with each of the 10 pre-specified medical, psychiatric and substance use conditions. The x-axis represents the 10 medical, psychiatric and substance use conditions and the y-axis represents the percent of subjects in each group with that condition. In Group 1, prevalence of medical, psychiatric and substance use conditions was relatively low compared to the four other groups. Group 2 had the highest prevalence of chronic pulmonary disease and anxiety disorders, and high prevalence of cardiovascular, end organ disease, diabetes, and mood disorders despite relatively lower prevalence of substance use disorders. Group 3 had the highest prevalence of end organ disease, opioid and stimulant use disorders, and mood disorders as well as a relatively high prevalence of all other conditions. Group 4 had the highest prevalence of alcohol use and psychotic disorders, and Group 5 had the highest prevalence of cardiovascular disease and diabetes.

Based on these findings, we named Group 1 [the referent group; *n* = 100 (20.1%)] “Low morbidity,” Group 2 [*n* = 102 (20.5%)] “high comorbidity,” Group 3 [*n* = 127 (25.6%)] “high tri-morbidity,” Group 4 [*n* = 56 (11.3%)] “high alcohol use,” and Group 5 [*n* = 112 (22.5%)] “high medical illness.”

### Demographics and Clinical Characteristics of Population

Of the 497 patients included in this study, the mean age was 50.5 (±11.0) years, 68.6% were male, and 61.1% were Black. A total of 36.8% of patients were uninsured, 40.6% had public insurance, and 2.4% had private insurance. For the entire cohort, the prevalence of (a) cardiovascular disease was 75.9%, (b) chronic pulmonary disease was 36.2%, (c) other end organ disease was 36.4%, and (d) diabetes was 32.4%. Alcohol use disorder was present in 38.8% of the cohort, and opioid use disorder and stimulant use disorder were present in 17.7 and 37.6% of the cohort, respectively. Finally, 13.9% of the cohort had a psychotic disorder, 50.9% had a mood disorder, and 43.5% had an anxiety disorder. Socio-economic and clinical characteristics varied across LCA groups. See Table 1 for complete demographic and clinical details. Supplementary 3 includes odds ratios and confidence intervals for all risk factors across each class.

**Table 1:** Baseline demographic and clinical characteristics of patients referred to DHCT program.

**Characteristic *n* (%)**	**Group 1: Low morbidity, 100 (20.1)**	**Group 2: High comorbidity, 102 (20.5)**	**Group 3: High tri-morbidity, 127 (25.6)**	**Group 4: High alcohol use, 56 (11.3)**	**Group 5: High medical illness, 112 (22.5)**	**Overall, 497 (100)**	*P*-value^*^
**Demographic characteristic**							
**Sex**							
Female	34 (34.0)	52 (51.0)	44 (34.7)	9 (16.1)	17 (15.2)	156 (31.4)	<0.0001
Male	66 (66.0)	50 (49.0)	83 (65.4)	47 (83.9)	95 (84.8)	341 (68.6)	
**Age** in years, M (SD) at program DHCT program referral	50.6 (13.5)	50.4 (11.4)	48.2 (9.7)	48.5 (11.0)	53.9 (8.9)	50.5 (11.0)	0.001
**Race**							
Black/African American	52 (52.0)	58 (56.9)	79 (62.2)	35 (62.5)	80 (71.4)	304 (61.1)	0.001
White	28 (28.0)	39 (38.2)	40 (31.5)	16 (28.6)	23 (20.5)	146 (29.4)	
Other/missing	20 (20.0)	5 (4.9)	8 (6.3)	5 (8.9)	9 (8.0)	47 (9.5)	
Insurance status—^*^Encounter/fluid at referral or next encounter							
Public	34 (34.0)	60 (58.8)	52 (40.9)	14 (25.0)	42 (37.5)	202 (40.6)	<0.0001
Private	4 (4.0)	2 (2.0)	1 (0.8)	1 (1.8)	4 (3.6)	12 (2.4)	
Uninsured	50 (50.0)	24 (23.5)	43 (33.9)	32 (57.1)	34 (30.4)	183 (36.8)	
Mixed	12 (12.0)	16 (15.7)	31 (24.4)	9 (16.1)	32 (28.6)	100 (20.2)	
**Clinical characteristics**							
**Chronic medical condition**							
Cardiovascular disease^a^	34 (34.0)	92 (90.2)	110 (86.6)	31 (55.4)	110 (98.2)	377 (75.9)	<0.0001
Chronic pulmonary disease^b^	22 (22.0)	54 (52.9)	65 (51.2)	7 (12.5)	32 (28.6)	180 (36.2)	<0.0001
Other end organ disease^c^	1 (1.0)	46 (45.1)	70 (55.1)	10 (17.9)	54 (48.2)	181 (36.4)	<0.0001
Diabetes^d^	2 (2.0)	45 (44.1)	42 (33.1)	1 (1.8)	71 (63.4)	161 (32.4)	<0.0001
**Substance use disorder**							
Alcohol use disorder	0 (0.0)	32 (31.4)	85 (66.9)	56 (100.0)	20 (17.9)	193 (38.8)	<0.0001
Opioid use disorder	5 (5.0)	16 (15.7)	61 (48.0)	1 (1.8)	5 (4.5)	88 (17.7)	<0.0001
Stimulant use disorder	3 (3.0)	0 (0.0)	127 (100.0)	24 (42.9)	33 (29.5)	187 (37.6)	<0.0001
**Mental illness**							
Psychotic disorder	4 (4.0)	13 (12.8)	30 (23.6)	17 (30.4)	5 (4.5)	69 (13.9)	<0.0001
Mood disorder	6 (6.0)	82 (80.4)	110 (86.6)	26 (46.4)	29 (25.9)	253 (50.9)	<0.0001
Anxiety disorder	14 (14.0)	102 (100.0)	91 (71.7)	9 (16.1)	0 (0.0)	216 (43.5)	<0.0001

a *Cardiovascular disease = congestive heart failure, valvular disease, and cardiac arrhythmia, complicated hypertension, uncomplicated hypertension and peripheral vascular disease*.

b *Chronic pulmonary disease = COPD and pulmonary circ disorders*.

c *Other End organ disease = renal failure and liver disease*.

d *Diabetes*.

* *P-value is an omnibus 4 degree of freedom test for differences in distribution in characteristic between the 5 latent class groups*.

### Health Care Utilization at 12 Months After Referral

Groups 2–5 had more admissions, longer duration of admissions, and more ED visits in the 12 months following referral compared to the “low morbidity” group. All groups except the “high alcohol use” group had more outpatient visits in the 12 months after referral compared to the “low morbidity” group. In comparison with the “low morbidity” group, the “high tri-morbidity” group had the largest magnitude increase in admissions (RR, 1.93; 95% CI, 1.32–2.81; *p* = 0.0007) and in ED visits (RR, 3.58; 95% CI, 2.79–4.58; *p* < 0.0001), and the “high comorbidity” group had the largest magnitude increase in mean length of admissions (RR, 2.11; 95% CI, 1.86–2.39; *p* < 0.0001) and in number of outpatient visits (RR, 1.39; 95% CI, 1.26–1.52, *p* < 0.0001). Complete health care utilization data is presented in Table 2.

**Table 2:** Health care utilization by class using Zero Inflated Poisson (ZIP) analysis to estimate Relative Risk (RR) with Low Morbidity group as the referent.

	**ZIP**		
**Utilization**	**RR**	**95% CI**	***P*-value**
**Admissions (#) in 12 months**			
Group 1: Low morbidity (referent)	1.00	reference	n/a
Group 2: High comorbidity	1.88	1.28–2.77	0.0013
Group 3: High tri-morbidity	1.93	1.32–2.81	0.0007
Group 4: High alcohol use	1.48	0.94–2.31	0.09
Group 5: High medical illness	1.69	1.14–2.51	0.009
**Length of stay (mean)**			
Group 1: Low morbidity (referent)	1.00	reference	n/a
Group 2: High comorbidity	2.11	1.86–2.39	<0.0001
Group 3: High tri-morbidity	1.55	1.36–1.76	<0.0001
Group 4: High alcohol use	1.58	1.36–1.83	<0.0001
Group 5: High medical illness	1.56	1.37–1.78	<0.0001
**ED visits (#) in 12 months**			
Group 1: Low morbidity (referent)	1.00	Reference	n/a
Group 2: High comorbidity	1.57	1.20–2.04	0.001
Group 3: High tri-morbidity	3.58	2.79–4.58	<0.0001
Group 4: High alcohol use	1.99	1.50–2.63	<0.0001
Group 5: High medical illness	1.75	1.33–2.28	<0.0001
**Outpatient visits (#) 12 months**			
Group 1: Low morbidity (referent)	1.00	reference	n/a
Group 2: High comorbidity	1.39	1.26–1.52	<0.0001
Group 3: High tri-morbidity	1.15	1.05–1.26	0.003
Group 4: High alcohol use	1.04	0.92–1.16	0.55
Group 5: High medical illness	1.35	1.23–1.48	<0.0001

### Mortality and Drug Overdose at 12 Months After Referral

Only the group with “high medical illness” had a greater mortality 12 months after referral compared to the “low morbidity” group (OR, 2.53, 1.03–6.16; 95% CI, 1.03–6.16; *p* = 0.04). Both the “high comorbidity” group (OR, 5.23; 95% CI, 1.57–17.39; *p* = 0.007) and “high tri-morbidity” group (OR, 4.20; 95% CI, 1.26–14.01; *p* = 0.02) had a greater 12-month drug overdose risk after referral compared to the referent group. Complete clinical outcome data are present in Table 3.

**Table 3:** Clinical outcomes by class using Logistic Regression to estimate Odds Ratios with Low Morbidity group as the referent.

**Clinical outcome**	**OR**	**95% CI**	***P*-value**
**Mortality at 12 months**			
Group 1: Low morbidity (referent)	1.00	reference	n/a
Group 2: High comorbidity	1.63	0.62–4.33	0.32
Group 3: High tri-morbidity	2.10	0.85–5.17	0.11
Group 4: High alcohol use	0.30	0.03–2.41	0.25
Group 5: High medical illness	2.53	1.03–6.16	0.04
**Drug overdose at 12 months**			
Group 1: Low morbidity (referent)	1.00	reference	n/a
Group 2: High comorbidity	5.23	1.57–17.39	0.007
Group 3: High tri-morbidity	4.20	1.26–14.01	0.02
Group 4: High alcohol use	1.19	0.21–6.91	0.84
Group 5: High medical illness	2.68	0.75–9.62	0.13

## Discussion

This study contributes to the body of literature examining utilization and comorbidity patterns among PEH. In this LCA of 497 individuals referred to a homeless transitional care program, we used a data-driven, probability-based method to classify the cohort into five distinct groups. To our knowledge, this is the first study to use EHR data that included medical, psychiatric and substance use diagnoses to build an LCA of PEH and to assess their health care utilization and clinical outcomes.

In regard to healthcare utilization, all groups in our study had significantly greater number of admissions, longer mean duration of admissions, and more ED visits in the 12 months after referral compared to the referent “low morbidity” group. These results align with those of prior latent group analyses that have used claims data (17), survey data (14–16), or administrative data (18, 27) to demonstrate increased health services utilization among PEH. These findings further add to previous LCAs that have described subgroups of heterogeneous homeless populations based on social characteristics (28) or patterns of shelter use (29, 30).

Our analysis supports similar findings of variable health care utilization among certain subgroups of PEH. Existing evidence indicates high ED utilization among subgroups of PEH with mental health disorders but without physical health disorders (16). Persistent hospital super-utilizers have been identified among subgroups with either mental health or substance use disorder (31). Among homeless subgroups, co-occurring mental health and substance use disorder drive increased ED and non-ED health care utilization, and persons with tri-morbid illness are among the costliest utilizers overall (17).

Concerning clinical outcomes, the “high medical illness” group in our analysis had greater mortality 12 months after referral compared to the “low morbidity” group. This is consistent with the existing literature that chronic medical conditions are risk factors for death in PEH (32, 33) and suggests that PEH with chronical medical conditions are a particularly vulnerable subgroup in this and similar cohorts. Our analysis also demonstrated the “high comorbidity” and “high tri-morbidity” groups had greater 12-month drug overdose risk after referral compared to the referent group. This in line with data demonstrating that drug overdose is an epidemic among PEH (34) and supports literature demonstrating rising rates of tri-morbidity among PEH (35).

## Limitations

The current study was retrospective and included data from a single health system, which may limit generalizability. The cohort was identified based on referral to a homeless transitional care program and therefore does not capture PEH who were not referred, limiting generalizability to the entire population experiencing homelessness. The observation period was limited to 12 months and data were limited with respect to patterns of homelessness among the study cohort. Finally, LCA carries inherent limitations, including risk of class misassignment, inability to determine exact number of members in classes because group membership is based on probabilities, and potential for “naming fallacy,” where class names do not accurately reflect the latent classes (8).

## Conclusion

This study describe how distinct groups of PEH are affected differently by comorbidities and supports the need for integrated medical, mental health, and substance use disorder services in programs that serve PEH.

## Data Availability Statement

The original contributions presented in the study are included in the article/Supplementary Material, further inquiries can be directed to the corresponding authors.

## Ethics Statement

The study was reviewed and approved by the Duke Health Institutional Review Board. Written informed consent for participation was not required for this study in accordance with the national legislation and the institutional requirements.

## Author Contributions

CS, JF, RS, and DB conceptualized the study. RS conducted the data analysis. All authors contributed to manuscript preparation and review and approved the submitted version of the article.

## Funding

This project is included as part of the Duke School of Medicine Opioid Collaboratory portfolio, grant-funded by the Duke Endowment and administered through the Duke Department of Population Health Sciences.

## Author Disclaimer

The opinions expressed in the article are the authors' own and do not necessarily reflect the views of Duke University, the US Government, or any agency thereof.

## Conflict of Interest

The authors declare that the research was conducted in the absence of any commercial or financial relationships that could be construed as a potential conflict of interest.

## Publisher's Note

All claims expressed in this article are solely those of the authors and do not necessarily represent those of their affiliated organizations, or those of the publisher, the editors and the reviewers. Any product that may be evaluated in this article, or claim that may be made by its manufacturer, is not guaranteed or endorsed by the publisher.

## References

[1] Kertesz SG Posner MA O'Connell JJ Swain S Mullins AN Shwartz M. Post-hospital medical respite care and hospital readmission of homeless persons. J Prev Interv Community. (2009) 37:129–42. 10.1080/10852350902735734 19363773 PMC2702998

[2] Bring C Kruse M Ankarfeldt MZ Brünés N Pedersen M Petersen J. Post-hospital medical respite care for homeless people in Denmark: a randomized controlled trial and cost-utility analysis. BMC Health Serv Res. (2020) 20:508. 10.1186/s12913-020-05358-4 32503545 PMC7275557

[3] Doran KM Ragins KT Gross CP Zerger S. Medical respite programs for homeless patients: a systematic review. J Health Care Poor Underserved. (2013) 24:499–524. 10.1353/hpu.2013.0053 23728025

[4] Fazel S Geddes JR Kushel M. The health of homeless people in high-income countries: descriptive epidemiology, health consequences, and clinical and policy recommendations. Lancet. (2014) 384:1529–40. 10.1016/S0140-6736(14)61132-6 25390578 PMC4520328

[5] Cheung A Somers JM Moniruzzaman A Patterson M Frankish CJ Krausz M. Emergency department use and hospitalizations among homeless adults with substance dependence and mental disorders. Addict Sci Clin Pract. (2015) 10:17. 10.1186/s13722-015-0038-1 26242968 PMC4636835

[6] Zhang L Norena M Gadermann A Hubley A Russell L Aubry T. Concurrent disorders and health care utilization among homeless and vulnerably housed persons in canada. J Dual Diagn. (2018) 14:21–31. 10.1080/15504263.2017.1392055 29494795

[7] Vuik SI Mayer EK Darzi A. Patient segmentation analysis offers significant benefits for integrated care and support. Health Aff . (2016) 35:769–75. 10.1377/hlthaff.2015.1311 27140981

[8] Weller BE Bowen NK Faubert SJ. Latent class analysis: a guide to best practice. J Black Psychol. (2020) 46:287–311. 10.1177/009579842093093227492449

[9] Berlin KS Williams NA Parra GR. An introduction to latent variable mixture modeling (part 1): overview and cross-sectional latent class and latent profile analyses. J Pediatr Psychol. (2014) 39:174–87. 10.1093/jpepsy/jst084 24277769

[10] Yan S Seng BJJ Kwan YH Tan CS Quah JHM Thumboo J. Identifying heterogeneous health profiles of primary care utilizers and their differential healthcare utilization and mortality - a retrospective cohort study. BMC Fam Pract. (2019) 20:54. 10.1186/s12875-019-0939-2 31014231 PMC6477732

[11] Mowbray CT Bybee D Cohen E. Describing the homeless mentally ill: cluster analysis results. Am J Community Psychol. (1993) 21:67–93. 10.1007/BF00938208 8213647

[12] Adlaf EM Zdanowicz YM. A cluster-analytic study of substance problems and mental health among street youths. Am J Drug Alcohol Abuse. (1999) 25:639–60. 10.1081/ADA-100101884 10548440

[13] Goldstein G Luther JF Jacoby AM Haas GL Gordon AJ. A Taxonomy of medical comorbidity for veterans who are homeless. J Health Care Poor Underserved. (2008) 19:991–1005. 10.1353/hpu.0.0040 18677085

[14] Aubry T Klodawsky F Coulombe D. Comparing the housing trajectories of different classes within a diverse homeless population. Am J Community Psychol. (2012) 49:142–55. 10.1007/s10464-011-9444-z 21557093

[15] Bonin JP Fournier L Blais R. A typology of mentally disordered users of resources for homeless people: towards better planning of mental health services. Adm Policy Ment Health. (2009) 36:223–35. 10.1007/s10488-009-0206-2 19214733

[16] Fleury MJ Grenier G Cao Z Meng X. Typology of currently or formerly homeless individuals based on their use of health and social services. Community Ment Health J. (2021) 57:948–59. 10.1007/s10597-020-00693-6 32734310

[17] Mitchell MS León CLK Byrne TH Lin WC Bharel M. Cost of health care utilization among homeless frequent emergency department users. Psychol Serv. (2017) 14:193–202. 10.1037/ser0000113 28481604

[18] Tsai J Kasprow WJ Rosenheck RA. Latent homeless risk profiles of a national sample of homeless veterans and their relation to program referral and admission patterns. Am J Public Health. (2013) 103(Suppl. 2):S239–47. 10.2105/AJPH.2013.301322 24148048 PMC3969139

[19] Elixhauser A Steiner C Harris DR Coffey RM. Comorbidity measures for use with administrative data. Med Care. (1998) 36:8–27. 10.1097/00005650-199801000-00004 9431328

[20] Quan H Sundararajan V Halfon P Fong A Burnand B Luthi JC. Coding algorithms for defining comorbidities in ICD-9-CM and ICD-10 administrative data. Med Care. (2005) 43:1130–9. 10.1097/01.mlr.0000182534.19832.83 16224307

[21] Austin SR Wong Y-N Uzzo RG Beck JR Egleston BL. Why summary comorbidity measures such as the Charlson Comorbidity Index and Elixhauser score work. Med Care. (2015) 53:e65–72. 10.1097/MLR.0b013e318297429c 23703645 PMC3818341

[22] Gutwinski S Schreiter S Deutscher K Fazel S. The prevalence of mental disorders among homeless people in high-income countries: an updated systematic review and meta-regression analysis. PLoS Med. (2021) 18:e1003750. 10.1371/journal.pmed.1003750 34424908 PMC8423293

[23] Pribish A Khalil N Mhaskar R Woodard L Mirza AS. Chronic disease burden of the homeless: a descriptive study of student-run free clinics in Tampa, Florida. J Community Health. (2019) 44:249–55. 10.1007/s10900-018-0580-3 30324539

[24] Nylund KL Asparouhov T Muthén BO. Deciding on the number of classes in latent class analysis and growth mixture modeling: a Monte Carlo simulation study. Struct Equ Model. (2007) 14:535–69. 10.1080/10705510701575396

[25] Nagin DS Odgers CL. Group-based trajectory modeling in clinical research. Annu Rev Clin Psychol. (2010) 6:109–38. 10.1146/annurev.clinpsy.121208.131413 20192788

[26] Lanza ST Collins LM Lemmon DR Schafer JL. PROC LCA: a SAS procedure for latent class analysis. Struct Equ Model Multidiscip J. (2007) 14:671–94. 10.1080/10705510701575602 19953201 PMC2785099

[27] Kuhn R Culhane DP. Applying cluster analysis to test a typology of homelessness by pattern of shelter utilization: results from the analysis of administrative data. Am J Community Psychol. (1998) 26:207–32. 10.1023/A:1022176402357 9693690

[28] Altena AM Beijersbergen MD Vermunt JK Wolf JRLM. Subgroups of Dutch homeless young adults based on risk- and protective factors for quality of life: results of a latent class analysis. Health Soc Care Community. (2018) 26:e587–97. 10.1111/hsc.12578 29664216

[29] Chu CMT Moodie EEM Streiner DL Latimer EA. Trajectories of homeless shelter utilization in the at home/Chez Soi trial of housing first. Psychiatr Serv. (2020) 71:648–55. 10.1176/appi.ps.201900260 32264800

[30] Gleason K Barile JP Baker CK. Describing trajectories of homeless service use in Hawai'i using latent class growth analysis. Am J Community Psychol. (2017) 59:158–71. 10.1002/ajcp.12128 28295354

[31] Szymkowiak D Montgomery AE Johnson EE Manning T O'Toole TP. Persistent super-utilization of acute care services among subgroups of veterans experiencing homelessness. Med Care. (2017) 55:893–900. 10.1097/MLR.0000000000000796 28863030

[32] Roncarati JS O'Connell JJ Hwang SW Baggett TP Cook EF Krieger N. The use of high-risk criteria to assess mortality risk among unsheltered homeless persons. J Health Care Poor Underserved. (2020) 31:441–54. 10.1353/hpu.2020.0032 32037341 PMC7376969

[33] Aldridge RW Story A Hwang SW Nordentoft M Luchenski SA Hartwell G. Morbidity and mortality in homeless individuals, prisoners, sex workers, and individuals with substance use disorders in high-income countries: a systematic review and meta-analysis. Lancet. (2018) 391:241–50. 10.1016/S0140-6736(17)31869-X 29137869 PMC5803132

[34] Baggett TP Hwang SW O'Connell JJ Porneala BC Stringfellow EJ Orav EJ. Mortality among homeless adults in Boston: shifts in causes of death over a 15-year period. JAMA Intern Med. (2013) 173:189–95. 10.1001/jamainternmed.2013.1604 23318302 PMC3713619

[35] Vickery KD Winkelman TNA Ford BR Busch A Robertshaw D Pittman B. Trends in trimorbidity among adults experiencing homelessness in minnesota, 2000-2018. Med Care. (2021) 59(Suppl. 2):S220–7. 10.1097/MLR.0000000000001435 33710099 PMC7958979

