# Supplementary material for: Differences in Clinical Outcomes of Adults Referred to a Homeless Transitional Care Program Based on Multimorbid Health Profiles: A Latent Class Analysis
Source: Front Psychiatry. 2021 Dec 20;12:780366. doi: 10.3389/fpsyt.2021.780366 (PMC8721199; doi:10.3389/fpsyt.2021.780366)
Supplement: Supplementary file 1 [file Data_Sheet_1.docx]

**Supplement 1**. Diagnostic codes for mental illness and substance use disorders

| Diagnosis | ICD-10-CM codes |
| --- | --- |
| Mental Illness | |
| Psychotic disorder | F20, F22-F29 |
| Mood | F30, F31, F34.0, F32, F33, F34.1 |
| Anxiety disorder | F40-F42,F43 |
| Substance use disorder | |
| Alcohol | F10 |
| Opioid | F11 |
| Stimulant | F14, F15 |
| Medical illness | |
| Cardiovascular | A520, I05-I08, I11-I13, I15, I34-I39, I43, I47-I50, I70, I71, I091, I098, I099, I110, I130, I132, I255, I420, I425-I429, I441-I443, I456, I459, I731, I738, I739, I771, I790, I792, K551, K558, K559, P290, Q230, Q231-Q233, R000, R001, R008, T821, Z450, Z950, Z952-Z954, Z958, Z959 |
| Diabetes | E100, E101, E109-E111, E119-E121, E129-E131, E139-E141, E149, E102-E108, E112-E115-E118, E122-E128, E132-E138, E142-E148 |
| Other end organ disease | B18, I85, I120, I131, I864, I982, K70, K72-K74, K760, K762-K769, K711, K713-K715, K717, N18, N19, N250, Z490-Z492, Z940, Z992, Z944 |
| Chronic pulmonary disease | I26, I27, I278-I280, I288, I289, J40, J41-J47, J60-J67, J684, J701, J703 |

**Supplement 2a:** Proc LCA to estimate number of latent classes DCHP Referral Subjects

|  | Percent of subjects assigned to each latent class | | | | | |  |
| --- | --- | --- | --- | --- | --- | --- | --- |
| Number latent classes | BIC | 1 | 2 | 3 | 4 | 5 | 6 |
| 1 | 1159.56 | 100 |  |  |  |  |  |
| 2 | 878.81 | 54.0 | 46.0 |  |  |  |  |
| 3 | 841.02 | 25.7 | 34.0 | 40.3 |  |  |  |
| 4 | 805.74 | 21.4 | 20.2 | 31.3 | 27.1 |  |  |
| 5 | 784.60 | 20.7 | 18.4 | 24.6 | 11.8 | 24.4 |  |
| 6 | 784.21 | 15.8 | 15.0 | 27.1 | 4.3 | 26.6 | 11.3 |

Entropy for 5 class solution: 0.87

**Supplement 2b:** LCA posterior probability among classes

| Group | N Obs | Variable | Label | Mean | Std | Minimum | Maximum |
| --- | --- | --- | --- | --- | --- | --- | --- |
| 1 | 102 | POSTCL1 | Latent Class 1 Posterior Prob | 0.89 | 0.13 | 0.69 | 0.99 |
| 2 | 100 | POSTLC2 | Latent Class 2 Posterior Prob | 0.92 | 0.10 | 0.79 | 0.99 |
| 3 | 127 | POSTCL3 | Latent Class 3 Posterior Prob | 0.87 | 0.11 | 0.72 | 1.00 |
| 4 | 56 | POSTLC4 | Latent Class 4 Posterior Prob | 0.88 | 0.10 | 0.75 | 0.99 |
| 5 | 112 | POSTLC5 | Latent Class 5 Posterior Prob | 0.95 | 0.15 | 0.71 | 1.00 |

**Supplement 3:** Odds Ratios (OR) by LCA class for each of the 10 conditions included in the Latent Class modeling.

| characteristic | Group 1: Low morbidity  (n = 100) | Group 2:  High comorbidity  (n = 102) | *Group 3: High tri-morbidity  (n = 127) | Group 4: High alcohol use  (n = 56) | Group 5:  High medical illness  (n = 112) |
| --- | --- | --- | --- | --- | --- |
| Chronic medical condition |  |  | REF |  |  |
| Cardiovascular disease^1^ | 0.08 (0.04, 0.15) / <.0001 | 1.42 (0.62, 3.26) / 0.40 | REF | 0.19 (0.09, 0.40) /<.0001 | 8.50 (1.92, 37.6) / 0.005 |
| Chronic pulmonary disease ^2^ | 0.27 (0.15, 0.48) / <.0001 | 1.07 (0.64, 1.81) / 0.79 | REF | 0.14 (0.07, 0.32) / <.0001 | 0.38 (0.22, 0.65) / 0.0004 |
| Other end organ disease^3^ | 0.01 (0.00, 0.06) / <.0001 | 0.67 (0.40, 1.13) / 0.13 | REF | 0.08 (0.08, 0.38) /<.0001 | 0.76 (0.45, 1.26) / 0.27 |
| Diabetes^4^ | 0.04 (0.01, 0.18) / <.0001 | 1.60 (0.93, 2.74) / 0.09 | REF | 0.04 (0.01, 0.28) / 0.001 | 3.51 (2.06, 5.98) / <.0001 |
| Substance use disorder |  |  |  |  |  |
| Alcohol use disorder | Non-est (zero cell) | 0.23 (0.13,0.40) /<..0001 | REF | Non-est (100%) | 0.11 (0.07, 0.20 /<..0001 |
| Opioid use disorder | 0.07 (0.02, 0.15) /<.0001 | 0.20 (0.11, .38) / <.0001 | REF | 0.02 (0.00, 0.15) /0.001 | 0.05 (0.02, 0.13) /<.0001 |
| Stimulant use disorder |  |  | REF |  |  |
| Mental Illness |  |  |  |  |  |
| Psychotic disorder | 0.14 (0.05, 0.40) / 0.003 | 0.47 (0.23, 0.96) / 0.04 | REF | 1.41 (0.70, 2.84) /0.34 | 0.15 (0.06, 0.41) / 0.002 |
| Mood disorder | 0.01 (0.00, 0.03) / <.0001 | 0.63 (0.31, 1.29) / 0.20 | REF | 0.13 (0.06, 0.28) / <.0001 | 0.06 (0.03, 0.11) / <.0001 |
| Anxiety disorder | 0.06 (0.03, 0.13) / <.0001 | Non-est | REF | 0.08 (0.03, 0.17) / <.0001 | Non-est |

*Reference group is the High tri-morbidity group, due to it having the largest number of individuals. Note that some of the ORs were non-estimable due to the low occurrence.
